# A phase I/II trial of irinotecan–cisplatin combined with an anti-late-diarrhoeal programme to evaluate the safety and antitumour response of this combination therapy in patients with advanced non-small-cell lung cancer

**DOI:** 10.1038/sj.bjc.6602866

**Published:** 2005-11-15

**Authors:** Y Takeda, E Tsuduki, S Izumi, M Hojo, M Kamimura, G Naka, K Kobayashi, K Kudo

**Affiliations:** 1 Department of Respiratory Medicine, International Medical Center of Japan, 1-21-1 Toyama Shinjuku-ku, Tokyo 162-8655, Japan; 2 Department of Respiratory Medicine, Saitama Cancer Center, Komuro, Ina 362-0806, Saitama, Japan

**Keywords:** irinotecan, anti-late-diarrhoeal program, cisplatin, non-small-cell lung cancer

## Abstract

We conducted a phase I/II study in patients with advanced non-small-cell lung cancer (NSCLC) to increase the therapeutic index of the cisplatin–irinotecan combination by institution of an anti-late-diarrhoeal program (ADP). A total of 77 chemotherapy-naive patients with advanced NSCLC were enrolled. The cisplatin dose was fixed at 60 mg m^−2^ (Day 1). Irinotecan was escalated in 5 mg m^−2^ increments, starting from 60 mg m^−2^ (Days 1 and 8). ADP consisted of oral sodium bicarbonate, magnesium oxide, basic water, and ursodeoxycholic acid, and was administered orally for 4 days with each dose of irinotecan. In the phase I portion, irinotecan pharmacokinetics was also examined. After the recommended dose of irinotecan with ADP was determined, a phase II study was conducted to evaluate the response. Maximum tolerated dose was reached at an irinotecan dose of 80 mg m^−2^ (Grade 4 diarrhoea and neutropenia). Pharmacokinetic studies show that the maximum concentration and the area under the curve of both irinotecan and SN38 (active metabolite of irinotecan) tend to increase in the dose-dependent manner of irinotecan. The phase II portion of the study included 48 patients, who were treated with 75 mg m^−2^ of irinotecan. Grade 3/4 toxicities included neutropenia in 65%, leucopenia in 33%, and late diarrhoea in 6% of the patients. During this treatment, PS did not change in 65% of patients. At the end of the chemotherapy, PS did not decline in 90% of patients. In the phase II portion, a response occurred in 63% (95% confidential interval (CI), 47–76%) of patients. Median time to progression was 19 weeks (95% CI, 15–22 weeks), and median survival was 52 weeks (95% CI, 39–64 weeks). This regimen of irinotecan and cisplatin with ADP resulted in promising efficacy with acceptable toxicity for patients with advanced NSCLC. This regimen is a candidate for the experimental arm towards future phase III studies.

Lung cancer is a major health-care problem worldwide. Patients with advanced non-small-cell lung cancer (NSCLC) have a poor prognosis because this disease is incurable with currently available treatments. Platinum-based two drug combinations improve survival as well as the quality of life for patients with advanced NSCLC (Non-small Cell Lung Cancer Collaborative Group, 1995). However, an efficacy plateau has been reached with the currently available two-drug combinations in the treatment of advanced NSCLC (Schiller *et al*, 2002). Clinical trials are ongoing to investigate novel combination chemotherapies to improve the outcome for advanced NSCLC.

Irinotecan, a semisynthetic derivative of the plant alkaloid camptothecin, exhibits antitumour activity by inhibiting topoisomerase I. Irinotecan has been approved for anticancer therapy in the United States, Europe, and Japan (Garcia-Carbonero and Supko, 2002). However, irinotecan is associated with toxicities, including severe late diarrhoea and leucopenia (Fukuoka *et al*, 1992; Kobayashi *et al*, 1998). Some studies have documented success with high-dose loperamide for treating irinotecan-associated late diarrhoea (Bleiberg and Cvitkovic, 1996; Merrouche *et al*, 1997). However, there is still no effective strategy for preventing this dose-limiting adverse effect. Grade 3/4 late diarrhoea occurs in 20–30% of patients treated with irinotecan at the recommended doses (Fukuoka *et al*, 1992; Conti *et al*, 1996; Saltz *et al*, 2000). Therefore, strategies to prevent late diarrhoea should allow for more effective use of this drug.

Alkaline conditions within the intestinal lumen decrease reabsorption of irinotecan and its metabolites (Kobayashi *et al*, 1999; Ikegami *et al*, 2002). The rationale was to prevent absorption by oral alkalisation (OA), which should in turn reduce epithelial damage and its impact on subsequent late diarrhoea. Controlling defecation (CD) should prevent constipation, thereby also preventing epithelial disruption and allowing less time for additional absorption. Based on these concepts, we conducted a case–control study to evaluate the ability of OA and CD treatment to prevent irinotecan-induced side effects (Takeda *et al*, 2001). A multivariate analysis showed that OA and CD treatment reduced the incidence of side effects such as late diarrhoea, emesis, and leucopenia. Hence, we hypothesised that OA and CD could be used as an anti-late-diarrhoeal programme (ADP) with irinotecan therapy. We designed a novel regimen to increase the therapeutic index of the cisplatin-irinotecan combination by decreasing the toxicities of irinotecan and instituting ADP. The main objectives of this study were to determine the maximum tolerated dose of irinotecan and cisplatin with the use of ADP in the phase I portion of the study and to evaluate the response rate of irinotecan and cisplatin with ADP in chemotherapy-naive advanced NSCLC patients in the phase II portion of the study.

## PATIENTS AND METHODS

### Patients

All patients had histologically or cytologically confirmed NSCLC. Eligibility criteria included: (1) surgically unresectable, stage III or IV disease; (2) no prior chemotherapy; (3) age 20–75 years; (4) performance status (PS) ⩽2 on the Eastern Cooperative Oncology Group (ECOG) scale; (5) adequate bone marrow function (leucocyte count >4000 *μ*l^−1^; platelet count >100 000 *μ*l^−1^; haemoglobin concentration >9 g dl^−1^), hepatic function (bilirubin <1.5 mg ml^−1^; transaminases <2 × upper limit of normal), and renal function (creatinine <1.5 mg dl^−1^ and creatinine clearance >60 ml min^−1^); (6) life expectancy of 8 weeks or longer; (7) written informed consent; and (8) The presence of measurable disease was necessary in patients with only stage IIIB or IV disease who were enrolled in the phase II portion of the study.

Exclusion criteria were: (1) massive pleural effusion or cardiac effusion (cardiac tamponade); (2) symptomatic brain metastases; (3) prior surgery within 4 weeks; (4) uncontrolled comorbid diseases such as angina pectoris, cardiac infarction within 3 months, cardiac failure, uncontrolled hypertension, uncontrolled diabetes mellitus, active infectious diseases, ileus, interstitial pneumonitis, or lung fibrosis; (5) concurrent malignancies; (6) pregnancy or lactation; (7) inability to consume sodium and water as required by ADP regimen. This protocol was approved by the Institutional Review Board of the International Medical Center of Japan.

### Treatment

All patients were treated with combined chemotherapy consisting of cisplatin and irinotecan delivered every 3 weeks. Cisplatin (60 mg m^−2^) was administered intravenously on Day 1 after adequate hydration. On Days 1 and 8, irinotecan was given in 500 ml of normal saline as a 90 min intravenously (i.v.) infusion. All patients received the same antiemetic regimen, which consisted of metoclopramide (10–20 mg day^−1^ for 4 days), corticosteroid (4–5 mg day^−1^ betamethasone for 3 days), and 5-hydroxytryptamine (5-HT3) receptor antagonist (1 mg day^−1^ granisetron for 2 days) given prophylactically by i.v. infusion before and after the administration of chemotherapy. After that, oral administration of 5-HT3 antagonists (4 mg day^−1^ ondansetron) was continued for 4 days. The dose of irinotecan was withheld for leucopenia (<3000 *μ*l^−1^) and/or diarrhoea >Grade 1. Granulocyte-colony-stimulating factor (G-CSF) was administered when Grade 3 leucopenia (<2000 *μ*l^−1^) and/or granulocytopenia (<l000 *μ*l^−1^) were observed. Before the next cycle was started, the leucocyte and platelet counts had to be ⩾3000 and 100 000 *μ*l^−1^, respectively. If more than 6 weeks passed from the time of the last treatment before these criteria were satisfied, the patient was removed from the study.

The rationale for the ADP used in this study has been reported in more detail elsewhere (Takeda *et al*, 2001). In brief, ADP was started in the morning on Day 1 before irinotecan and cisplatin administration and continued for 4 days. ADP consisted of sodium bicarbonate, magnesium oxide, basic water, and ursodeoxycholic acid (UDCA) administered orally. The former three agents have a basic pH and directly mediate alkalisation from the duodenal lumen. Ursodeoxycholic acid has been reported to stimulate bile flow associated with a bicarbonate-rich choleresis and to increase biliary pH (Strazzabosco *et al*, 1991). Magnesium oxide demonstrates a laxative action, which was intended to shorten the dwelling time of irinotecan and its metabolites within the intestine. For OA, patients were given sodium bicarbonate 0.5 g orally and magnesium oxide 0.5** **g orally after every meal and before sleep, for a total of four doses per day. Patients also took UDCA 100 mg orally after every meal, for a total of three doses per day, and basic water (pH>7.2) for a total of 1500–2000 ml per day. Additional magnesium oxide and basic water were also given to patients to control defecation, with a goal of two bowel movements per day (CD). If patients developed watery diarrhoea during ADP, magnesium oxide administration was stopped. If diarrhoea occurred on the day when irinotecan was administered, anticholinergic drugs were used for treatment (Gandia *et al*, 1993). Late diarrhoea, which occurred more than 6 days after each dose of irinotecan administration, was treated with a high dose of loperamide as described by Bleiberg *et al* (Bleiberg and Cvitkovic, 1996). Since irinotecan was administered on Days 1 and 8, we defined late diarrhoea as occurring more than 6 days after irinotecan administration and not explained by ADP-induced early diarrhoea. ADP-induced early diarrhoea was defined by checking the pH of the stool. If diarrhoea pH was alkaline during the period of ADP, diarrhoea was thought to be from ADP (Takeda *et al*, 2001). If high-dose loperamide therapy did not stop late diarrhoea altogether or if late diarrhoea of ⩾Grade 3 appeared, the patient was given fluid by intravenous hyperalimentation (IVH).

### Study design

The doses of irinotecan were escalated in 5 mg increments from 60 mg m^−2^, using three to six patient cohorts (Table 2). The first dose level was 60 mg m^−2^ of irinotecan on Days 1 and 8 with no ADP (60N). For the second level (60P) and beyond, patients were treated with ADP. The maximum tolerated dose (MTD) was defined as the dose at which dose-limiting toxicities (DLTs) occurred in one-third, or more, of the patients. DLT was defined as: (1) ECOG common toxicity criteria (CTC) Grade 4 leucopenia or Grade 4 neutropenia lasting more than 5 days; (2) Grade 4 leucopenia or Grade 4 neutropenia with fever more than 38°C; (3) Grade 4 thrombocytopenia; (4) Grade 3 or 4 nonhaematological toxicity (except for alopecia, nausea and vomiting, or constipation) lasting more than 5 days; (5) Grade 2 diarrhoea lasting more than 7 days; or (6) Grade 3/4 diarrhoea. Once the MTD and the recommended dose were defined, patients were accrued to the phase II portion of the study. The primary end point of this phase II study was response rate and secondary end points included time to progression (TTP), survival and determination of toxicities.

### Evaluation

Pretreatment evaluation included: PS, chest radiograph, bone scintiscan, computed tomography of the head, chest, and abdomen, and fiberoptic bronchoscopy. Prior to each chemotherapy cycle, patients were subjected to a complete blood cell count (CBC) that included a differential count, serum chemistry for renal and hepatic functions, electrolyte analysis, urinalysis, and PS. complete blood cell count, serum chemistry, electrolyte analysis, urinalysis, chest radiographs, and PS were assessed at least once a week after the initial evaluation. PS was also checked at the end of each chemotherapy cycle. During the cycle of chemotherapy, the pH of diarrhoea was examined using a pH meter, HM-14P (TOA Electronics Ltd, Tokyo, Japan) (Takeda *et al*, 2001) or pH test sheet (Universal test paper or Brom-Thymol-Blue test paper; Advantec, Toyo Roshi Kaisha Ltd, Tokyo, Japan). Tumour response was classified in accordance with World Health Organization criteria (World Health Organization, 1979). ECOG-CTC was used to Grade toxicity. Duration for toxicities, measured in this study, refers to the time required for recovery to toxicity ⩽Grade 1. The adherence of ADP was the average of the compliance of water intake and drug intake. The compliance of water intake was defined as: (total amount of water consumed)/(amount of water intake prescribed). The compliance of drug intake was calculated as the percentage of compliance with medicines that were to be taken as scheduled for 3 days. Analysis was conducted on an intent to treat for toxicity and efficacy profile. Subtraction PS was to subtract the PS at certain time points of chemotherapy from the PS at the start of chemotherapy. The minus value of subtraction PS indicates declined PS. The plus value indicates improved PS. Analyses on the follow-up data were performed when more than two-thirds of the patients were dead or 12 months had passed since the enrollment of the last patients. Time to progression was defined as the time from the date of entry to the date of progressive disease, the last follow-up, or death without progression. The duration of survival was determined as the number of weeks from the date of entry to death or the last follow-up.

### Pharmacokinetics

Blood samples (5 ml) were obtained from the arm opposite the one used for chemotherapy infusion before the irinotecan infusion, at the end of infusion, and 1, 2, 4, 8, 24, 48, 72 h after completion of the infusion. Serial plasma samples were acquired on the first cycle from the patients. Sample collection started in each patient when irinotecan was administered on Day 1. The blood was centrifuged immediately, and the plasma obtained was stored at −80°C until analysis. Total plasma concentration of irinotecan, SN-38 (active metabolite of irinotecan, 7-ethyl-10-hydroxycamptothecin), and SN38 glucuronide (SN-38G) were measured using the high-performance liquid chromatography method previously described (Kurita and Kaneda, 1999). Maximum plasma concentrations (Cmax, *μ*g ml^−1^), elimination half-life (*T*_1/2_, hours), areas under the plasma-concentration time curve (AUC_0-*∞*_, *μ*g h^−1^ ml^−1^), and mean resident time (MRT, hours) of each compound were determined by noncompartment analysis using the computer program, WinNonLin Professional version 4.1 (Pharsight Corporation, Mountain View, CA, USA).

### Statistical analysis

We chose a target response probability for the regimen of 60% and lowest response rate of interest to be 40%. According to the Simon two-stage optimal design, a total of 46 patients were required to test this hypothesis in the phase II portion. At least seven major responses had to be observed in the first 16 assessable patients to continue enrollment to the 2nd stage of the study (with type 1 and 2 errors of 0.05 and 0.20, respectively) (Simon, 1989). Patients not assessable for response (because of treatment refusal or early treatment discontinuation for any reasons other than progression) were considered as nonresponders in calculation of the objective response rate. We also calculated the 95% exact confidential intervals (CIs) for response rate. Patients who received at least one cycle of chemotherapy were considered evaluable for response. Overall survival curves were estimated by the Kaplan–Meier method (Kaplan and Meier, 1958). According to the report by Schemper and Smith, median follow-up was estimated by reverse Kaplan–Meier method (Schemper and Smith, 1996). All analyses were performed using the Statistical Package for Social Sciences (SPSS) for Windows 10.0J.

## RESULTS

### Patients

Between June 1997 and August 2002, 77 patients were enrolled at the International Medical Center of Japan. In the phase I portion of the study, all patients were evaluable for toxicities. In the phase II portion, 48 patients were enrolled, and all were evaluable for toxicity. In all, 71 patients had either stage IIIB or IV disease. Six had stage IIIA disease. Patients' characteristics are summarised in Table 1.

In all, 21 patients were women, and the median age of all patients was 64 years (range, 36–75 years). Of patients, 93% had a PS of 0 or 1. Three patients had undergone prior surgical resection (palliative surgery). One patient had received prior palliative irradiation therapy to brain metastasis.

### DLTs and MTD

In all, 29 patients received 61 cycles of chemotherapy in the phase I portion of the study (mean 2, range 1–4) (Table 2). There were no treatment-related deaths. Two of seven (29%) patients enrolled in dose level 60N experienced Grade 4 neutropenia lasting 6 and 7 days, respectively. In that the objective of this portion of the study was to determine the MTD for the combination with the use of ADP, dose escalation continued. There were no DLTs in patients treated in cohorts 60P and 65P. At 70P, only one of four patients (25%) exhibited Grade 3 toxicity (hyponatraemia for lasting 4 days, though it did not meet the definition of DLTs). At 75P, one of six patients (17%) experienced a DLT (Grade 3 serum glutamic oxaloacetic and glutamic pyruvic transaminases for 8 days). Two patients were enrolled in level 80P, and both of them experienced DLTs; one patient had Grade 4 leucopenia with fever, Grade 4 diarrhoea and Grade 3 delusions, and the other had Grade 4 diarrhoea and Grade 4 neutropenia with fever. Two consecutive patients also had severe toxicities that met DLTs. Even if an additional four patients were enrolled into this level, more than and equal to one-third of the patients experienced DLTs. The safety committee recommended not continuing the entry of this level. Therefore, the 80P was defined as the MTD and 75P was decided as the recommended dose for phase II studies. The adherence on ADP was more than 90% in patients treated in cohorts 60P to 75P, and all of the patients in those levels exhibited Grade 2 or less late diarrhoea. At the 80P dose level, severe nausea impaired adherence with ADP. Irinotecan dose actually delivered is listed in Table 2. Two patients (7%) could not be given irinotecan on Day 8, and three patients (10%) had its administration delayed by a few days. The median recovery period from side effects was 22 days (range, 17–30 days) in all patients enrolled onto the phase I portion of the study. G-CSF administration was required in 17% of the patients.

### Pharmacokinetics

**Figure 1:**
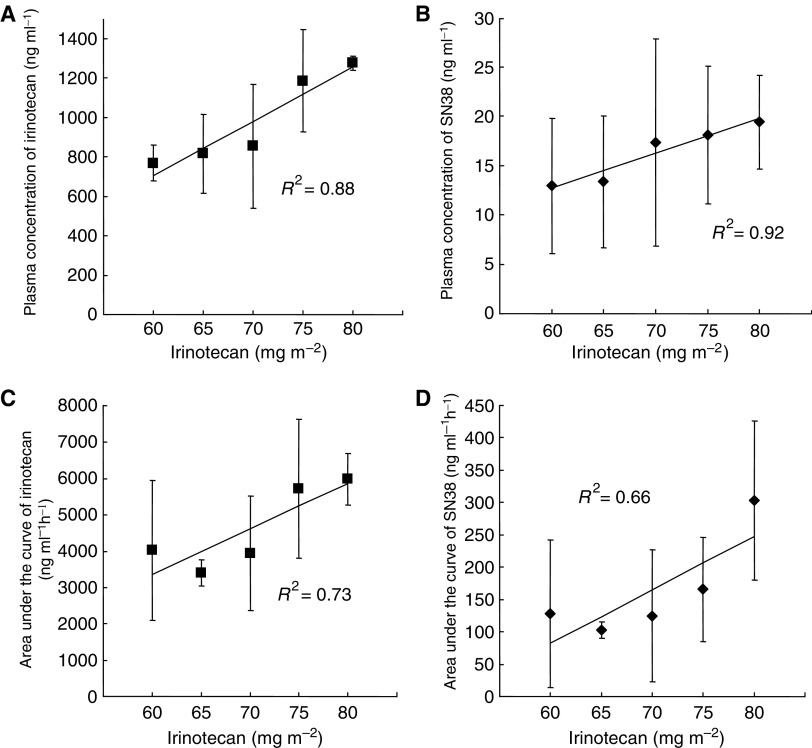
(**A**) Correlation between irinotecan dose (mg m^−2^) and maximum plasma concentration of irinotecan (*R*^2^=0.88, *P*=0.018). (**B**) Correlation between irinotecan dose (mg m^−2^) and maximum plasma concentration of SN38 (*R*^2^=0.92, *P*=0.01). (**C**) Correlation between irinotecan dose (mg m^−2^) and AUC_0-infinite_ of irinotecan (*R*^2^=0.73, *P*=0.067). (**D**) Correlation between irinotecan dose (mg m^−2^) and AUC_0-infinite_ of SN38 (*R*^2^=0.66, *P*=0.095).

Pharmacokinetic studies were performed in 17 patients receiving the phase I portion from 60P to 80P (Table 3). The Cmax and AUC of both irinotecan and SN38 tend to increase in a dose-dependent manner (Figure 1). The correlations between the administrated dose of irinotecan and the Cmax of both irinotecan and SN38 are also observed by simple regression models. However, no tendencies were observed in *T*_1/2_ and MRT of irinotecan. There is no consistent tendency in T_1/2_ and MRT of SN38 and in all parameters of SN38G.

### Toxicities in Phase II

In all, 48 patients and 140 cycles of chemotherapy (mean 3, range 1–5) were given in the phase II portion of the study. Table 2 shows the adherence of the therapy, recovery period, and additional supportive procedures by patient and cycle. When we evaluated these factors by patient, the worst values during the treatment were shown. The median adherence with ADP was 87% per patient (Table 2). Reduction of the irinotecan dose was performed in only two patients. The Day 8 dose of irinotecan could not be administered in 13%, whereas in 19 patients (40%) it was delayed. The median recovery period from all side effects was 26 days (range, 17–42 days). The toxicity data by patient and cycle are described in Tables 4 and 5. The maximum toxicities during the treatment are shown in terms of the data by patient. Grade 2 or greater leucopenia occurred in 37 patients (77%), with Grades 3 and 4 leucopenia occurring in 15 patients (31%) and 1 patient (2%), respectively (Table 4). Neutropenia ⩾Grade 2 was observed in 42 patients (88%), with Grades 3 and 4 neutropenia observed in 24 (50%) and seven (15%) patients, respectively. Febrile neutropenia, defined as Grade 3 or 4 neutropenia concomitant with ⩾Grade 2 fever, was observed in 11 patients (23%). As a result, 13 patients (27%) required G-CSF administration. Grade 3 or 4 anaemia was observed in nine patients (19%), and three patients (6%) required red blood cell transfusions. Grade 3 or greater thrombocytopenia was observed in only one patient. No patient required a platelet transfusion.

Gastrointestinal toxicity was the most prominent nonhaematological toxicity (Table 5). Nausea (>Grade 1) was the most common gastrointestinal toxicity, occurring in 65% of patients. According to the protocol, ADP was not discontinued if Grade 1 diarrhoea occurred. Since early diarrhoea included diarrhoea possibly related to ADP, the frequency of >Grade 1 was 38%. Only one patient had Grade 3 early diarrhoea, and she recovered from it when ADP was stopped. Furthermore, late diarrhoea >Grade 1 was observed in 10 (21%) patients. Grade 3 diarrhoea was observed in three patients (6%), and no Grade 4 diarrhoea was observed. Other Grade 3 or 4 nonhaematological toxicities were as follows: fatigue (two patients), infection except febrile neutropenia (one patient), liver dysfunction (toxicities of bilirubin, four patients; toxicities of transaminases, six patients), and hyponatraemia (one patient). There was no severe pulmonary toxicity noted in this study.

**Figure 2:**
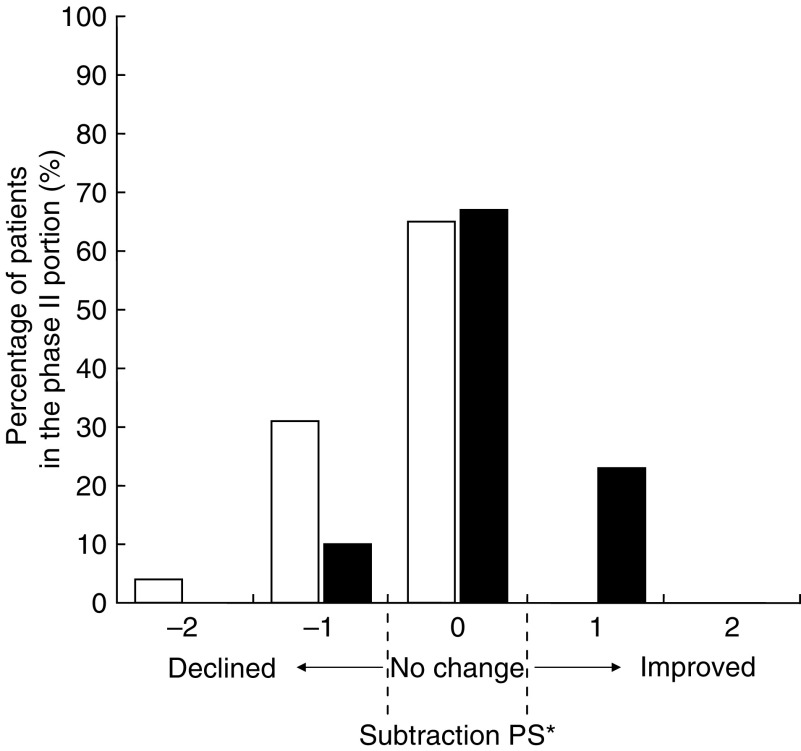
Change of performance status (PS) in each patient. ^*^; Subtraction PS is to subtract the PS at particular time points during chemotherapy from the PS at the start of the chemotherapy. Plus value of subtraction PS means improved PS. Minus value of subtraction PS means declined PS. Open bars represent subtraction PS during chemotherapy, which is to subtract the worst PS during total cycles in each patient from the PS at the start of chemotherapy. Solid bars represent subtraction PS at the end of the chemotherapy, which is to subtract the PS at the end of the treatment from the PS at the start of chemotherapy

In general, PS represents a common (albeit global) parameter of change (Cella *et al*, 1993). In the phase II portion, PS was serially checked during all cycles of the chemotherapy in each patient (Figure 2). The subtraction PS during chemotherapy by patient was calculated to subtract the worst PS during total cycles of chemotherapy from the PS at the start of chemotherapy. Two (4%) had a −2 value of subtraction PS during chemotherapy and 15 (31%) patients had a −1 value. Although 35% of patients had declined PS, 65% of patients had no change in PS during this treatment. The subtraction PS at the end was to subtract the PS at the end of this regimen from the PS at the start of chemotherapy. Five (10%) patients had a −1 value of subtraction PS at the end. In all, 11 (23%) patients had a +1 value. At the end of the chemotherapy, 23% of patients had improved PS and 67% of patients had no change in PS.

### Efficacy

A total of 27 patients were evaluable for response in the phase I portion of the study and all 48 of the patients in the phase II portion (Table 6). In the phase II portion of the study, the response rate was 63% (95% confidential interval (CI), 47–76%) by intent to treat analysis. Among these 75 evaluable patients, one patient had a complete response, 39 patients had a partial response, 23 patients had stable disease, and 12 patients had progressive disease. The overall response rate was 53% (95% CI, 41–65%).

As of November 2003, median follow-up was 56 months (95% CI, 17–95 months). All other patients, except one patient in the phase I portion of this study, were followed up. Median TTP was 19 weeks (95% CI, 14–25 weeks). Survival rates (1 and 2 years) were 59% (95% CI, 46–71%) and 23% (95% CI, 13–35%), respectively. Median survival was 56 weeks (95% CI, 42–70 weeks).

**Figure 3:**
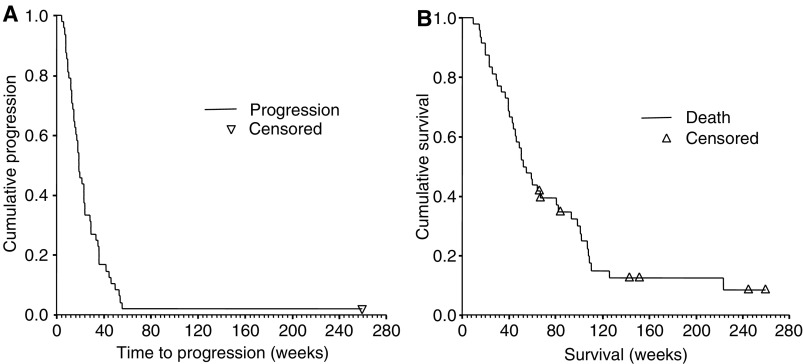
Time to progression and survival of 48 patients enrolled in phase II. (**A**) Time to progression of patients in phase II. (**B**) Survival of patients in phase II.

For the 48 patients in phase II (Figure 3), median TTP was 19 weeks (95% CI, 15–22 weeks, Figure 3a). One-year and two-year survival rates for the patients in phase II were 50% (95% CI, 35–65%) and 21% (95% CI, 10–35%), respectively. Median survival was 52 weeks (95% CI, 39–64 weeks, Figure 3b).

## DISCUSSION

Although the DLTs of single agent irinotecan include leucopenia and neutropenia, the principal toxicity is late diarrhoea. Its irinotecan-induced late diarrhoea appears unexpectedly and is unpredictable. We conducted a phase I/II study of irinotecan and cisplatin with the addition of ADP to decrease late diarrhoea. The first dose level of 60N is almost the same as the standard regimen used in Japan (Noda *et al*, 2002). The standard regimen of this combination chemotherapy consists of 60 mg m^−2^ cisplatin on Day 1 and 60 mg m^−2^ irinotecan on Days 1, 8, and 15, every 4 weeks. Grade 3 or 4 late diarrhoea occurred in 16% of their patients treated with cisplatin plus irinotecan and in none of their patients treated with cisplatin plus etoposide. We chose to study cisplatin in combination with irinotecan for the following reasons: (1) platinum-based chemotherapy is standard first-line treatment for advanced NSCLC; (2) the combination of cisplatin and irinotecan is associated with a high incidence of late diarrhoea. (3) Toxicity limited the delivery of the planned dose of irinotecan dose to less than 59% of cycles (Masuda *et al*, 1998). The ability to deliver the planned doses of irinotecan should lead to improvement of the efficacy of this combination regimen. In our study, 97% of the planned dose irinotecan dose by cycle was actually administered (Table 2). We hypothesised that incorporation of ADP would improve the delivery of the planned dose of irinotecan dose by decreasing side effects although we were aware of the possibility that altered metabolic clearance of irinotecan could result in reduced efficacy (Mathijssen *et al*, 2001). Even if this were the case, ADP could increase the actual usage of irinotecan. When the administration dose of irinotecan was increased by use of ADP, the AUC and the Cmax of irinotecan also increased (Figure 1). The results of our pharmacokinetic study show that the AUC and the Cmax of both irinotecan and SN38 tend to increase in the dose-dependent manner of irinotecan. Related to this phase I/II study, we also conducted another pharmacokinetic study to compare PK parameters with or without ADP. The analysis is ongoing using the noncompartment model. This preliminary data is showing that MRT of irinotecan and *T*_1/2_ of SN38 in high adherence of ADP were shorter than those in its low adherence. We supposed that the dose escalation of irinotecan could be performed by increasing the elimination of these two compounds.

There is one report in a phase II study of irinotecan and cisplatin without ADP, that grade 3 or 4 leucopenia, neutropenia, and late diarrhoea occurred in 32 (46%), 53 (80%), and 13 (19%), respectively, of 69 patients (Masuda *et al*, 1998). In the phase II portion of our study, grade 3 or 4 leucopenia, neutropenia, and late diarrhoea occurred in 33, 65, and 6% of patients, respectively (Tables 4 and 5). Even though their dose schedule was slightly different from ours, all of their major toxic effects are lower in our study than those in the above study. When we examined toxicities profile by cycle, grade 3 or 4 leucopenia, neutropenia, and late diarrhoea occurred in 17, 37, and 2% of cycles, respectively (Tables 4 and 5). A common parameter of change is PS and it was predicted that the physical and functional factor would show the most significant sensitivity to change in PS (Cella *et al*, 1993). In case of FACT-G, change in PS was related to physical, functional, and emotional factors but not to the social or relational situation with the doctor. In the EORTC QLQ-C30, the same relationship was observed (Aaronson *et al*, 1993). Hence, PS was serially checked during all cycles of this treatment by patients. Although 35% of patients temporally had declined PS probably due to the side effects, the PS did not change in 65% of patients during this treatment (Figure 2). At the end of the chemotherapy, PS had not declined in 90% of patients. These changes of PS might be feasible for patients who received the cisplatin-based doublet chemotherapy (Aaronson *et al*, 1993).

The response rate in our study was 63% in the phase II portion. Median TTP was 19 weeks and median survival 52 weeks. When we reviewed the patients enrolled in this study, there were two patients with stage IIIA disease in the phase II portion. However, in order to maintain the statistical power of this study, these two patients could not be removed from the efficacy profile. In order to compare the efficacy profiles with the other study, we also analysed the data of 46 patients with IIIB/IV. The response rate, median TTP, and median survival in them were 61% (95% CI, 45–75%), 18 weeks (95% CI, 15–21 weeks), and 52 weeks (95% CI, 40–65 weeks), respectively. There was a probability that these efficacy profiles might be better than those previously reported with other platinum-based combination regimens in phase II studies (Berthaud *et al*, 1992; Johnson *et al*, 1996; von Pawel *et al*, 1996; Masuda *et al*, 1998; Zalcberg *et al*, 1998; Bretti *et al*, 2002).

After this combination chemotherapy, 33 (69%) of 48 patients in our phase II were given second-line chemotherapies. Only 5 (10%) patients were treated by docetaxel (Shepherd *et al*, 2000). When these 5 patients were excluded, the median survival of 43 patients was 54 weeks (95% CI, 38–71 weeks). These data suggest that second-line chemotherapy by docetaxel might not influence the survival of patients in our study.

In conclusion, irinotecan and cisplatin can be made more tolerable with ADP. The irinotecan and cisplatin with ADP resulted in promising efficacy with acceptable toxicity. In order to prove the efficacy of this regimen, a randomised phase III trial is necessary.

## Figures and Tables

**Table 1:** Patients' characteristics

	**Phase**		
**Characteristics**	**I**	**II**	**Total**
*Sex (n)*			
Male	21	35	56
Female	8	13	21
			
*Age (years)*			
Median	66	62	64
Range	36–73	36–75	36–75
			
*ECOG PS (n)*			
0	10	10	20
1	16	36	52
2	3	2	5
			
*Histology (n)*			
Adenocarcinoma	25	35	60
Squamous cell carcinoma	3	9	12
Large cell carcinoma	1	4	5
			
*Prior treatment (n)*			
No	22	47	69
Yes	7	1	8
Surgery	6	1	7
Extrathoracic RT	1	0	1
			
*Stage (n)*			
IIIA	4	2	6
IIIB	4	12	16
IV	21	34	55

*n*, number of patients; RT, radiation therapy.

**Table 2:** Adherence and dose limiting toxicities (DLTs)

			**Irinotecan given on Day 8**						
**Dose level**	**Dose of irinotecan (mg m^−2^)**	**ADP^#1^ Adherence median (range, %)**	**Delay**	**Off**	**Delivered dose/planned dose of irinotecan (average, %)**	**Actual recovery period median (range, days)**	**With G-CSF**	**Blood transfusion**	**Patients with DLT**
Phase I (First cycle)									
60N	60	NE^#2^	0/7	0/7	100	22 (19–30)	2/7	0/7	2/7
60P	60	116 (82–140)	0/5	1/5^a^	90	22 (18–28)	1/5	0/5	0/5
65P	65	117 (91–123)	1/5^b^	0/5	100	22 (22–22)	0/5	0/5	0/5
70P	70	106 (104–107)	0/4	1/4^c^	88	22 (22–22)	0/4	0/4	0/4
75P	75	93 (82–101)	2/6^d^	0/6	100	21 (17–30)	1/6	0/6	1/6
80P	80	67 (58–76)	0/2	0/2	100	24 (23–24)	1/2	0/2	2/2
									
*Phase II (per patient)*									
	75	87 (64–104)	19/48	6/48	94	26 (17–42)	13/48	3/48	NE
									
*Phase II (all cycles)*									
	75	92 (64–121)	26/140	6/140	97	23 (17–42)	18/140	6/140	NE

ADP^#1^, anti-late-diarrhoeal programme; NE^#2^, not evaluated.

a Haematologic toxicity.

b Nonhaematologic toxicity (diarrhoea).

c Nonhaematologic toxicity (herpes zoster infection).

d Nonhaematologic toxicity (diarrhoea and liver dysfunction).

**Table 3:** Pharmcokinetic parameters of irinotecan, SN38, and SN38G in the phase I portion

**Dose level**	**60P**	**65P**	**70P**	**75P**	**80P**
No. Pts^*1^	**5**	**3**	**3**	**4**	**2**
	**Irinotecan**				
Cmax^*2^	768.0±89.62	816.7±200.3	853.3±313.4	1185±258.5	1275±35.36
T 1/2^*3^	9.69±2.26	13.8±4.36	21.2±1.72	13.8±1.82	11.5±3.22
AUC^*4^	4023±1919	3402±355.4	3947±1585	5719±1912	5987±703.1
MRT^*5^	5.13±1.40	5.67±2.12	9.42±0.50	6.18±1.31	6.51±0.42
					
	**SN38**				
Cmax	12.9±6.82	13.3±6.66	17.3±10.5	18.1±6.99	19.4±4.77
T 1/2	11.1±4.38	14.2±0.72	11.4±9.69	17.3±3.99	19.0±0.05
AUC	127.5±114	102.7±12.9	124.7±101.9	165.8±80.4	303.0±122.4
MRT	10.8±5.48	15.1±2.05	10.9±10.4	16.8±4.40	22.7±1.97
					
	**SN38G**				
Cmax	72.4±44.4	109.3±91.0	60.0±18.8	59.5±15.9	59.3±7.42
T 1/2	12.9±2.71	14.3±3.26	17.6±3.83	12.2±2.14	17.7±1.44
AUC	1007±751.6	1455±886.8	1086±182.4	993.7±561.6	1290±151.3
MRT	13.6±3.20	14.4±4.01	21.6±3.60	14.0±3.17	22.1±0.95

pts, patients; Cmax, maximun concentration (ng ml^−1^); *T*_1/2_, elimination half-life (h); AUC, area under the curve (ng ml^−1^ h^−1^); MRT, mean residence time (h).

**Table 4:** Haematologic toxicities

		**Number of patients (cycles) with toxicity**											
		**Leukopenia**				**Neutropenia**				**Anemia**		**Thrombocytopenia**	
**Dose level**	**No. Pts^a^ (cycles)**	**Mean nadir (Range: *μ*l^−1^)**	**Grade 2**	**Grade 3**	**Grade 4**	**Mean nadir (Range: *μ*l^−1^)**	**Grade 2**	**Grade 3**	**Grade 4**	**Mean nadir (Range: g dl^−1^)**	**Grade 3–4**	**Mean nadir (Range: × 10^4^ *μ*l^−1^)**	**Grade 3–4**
Phase I													
60N	7 (13)	2806 (1000–4160)	3 (5)	3 (3)	0	1276 (324–2288)	2 (5)	1 (2)	2 (2)	10.7 (8.0–12.7)	0	18.3 (11.3–25.1)	0
60P	5 (8)	2123 (1300–3560)	3 (4)	1 (3)	0	877 (312–1940)	1 (1)	2 (5)	1 (1)	9.8 (8.7–13.3)	0	14.8 (8.0–18.8)	0
65P	5 (14)	3055 (2100–4700)	4 (8)	0	0	1204 (483–1998)	2 (3)	2 (6)	1 (1)	10.7 (8.5–13.5)	0	15.9 (9.4–26.8)	0
70P	4 (9)	3167 (2000–4050)	1 (1)	0	0	1367 (860–1789)	1 (4)	3 (3)	0	11.4 (9.5–13.7)	0	23.4 (16.9–35.6)	0
75P	6 (15)	2825 (1800–4080)	4 (9)	1 (1)	0	1303 (378–2489)	3 (4)	2 (5)	1 (1)	9.8 (8.0–12.1)	0	20.6 (8.7–33.6)	0
80P	2 (2)	1235 (400–2070)	1 (1)	0	1 (1)	446 (200–691)	0	1 (1)	1 (1)	11.7 (10.8–12.5)	0	12.6 (9.4–15.8)	0
Phase II	48 (140)	2926 (510–7290)	21 (64)	15 (22)	1 (2)	1303 (112–3612)	11 (49)	24 (42)	7 (10)	9.8 (5.7–13.6)	9 (14)	19.2 (4.9–45.5)	1 (1)

a Number of patients (cycles).

**Table 5:** Nonhaematologic toxicities

	**Phase I**						
**Dose level**	**60N**	**60P**	**65P**	**70P**	**75P**	**80P**	**Phase II**
**Number of patients (cycles)**	**7 (13)**	**5 (8)**	**5 (14)**	**4 (9)**	**6 (15)**	**2 (2)**	**48 (140)**
*Fatigue*							
Grade 2	0	1 (1)	1 (1)	0	0	0	12 (16)
Grade 3	0	0	0	0	0	2 (2)	2 (2)
Grade 4	0	0	0	0	0	0	0
							
*Nausea*							
Grade 2	5 (8)	3 (3)	3 (6)	3 (5)	5 (10)	0	24 (40)
Grade 3	0	1 (1)	0	1 (1)	0	2 (2)	7 (10)
Grade 4	0	0	0	0	0	0	0
							
*Infection*							
Grade 2	1 (1)	0	1 (2)	2 (2)	1 (1)	0	9 (14)
Grade 3	1 (1)	0	0	0	0	0	1 (1)
Grade 4	0	0	0	0	0	0	0
+NPG^a^ ⩾3	1 (1)	0	0	0	1 (1)	2 (2)	11 (13)
							
*Early diarrhoea*							
Grade 2	1 (1)	1 (2)	1 (1)	1 (1)	3 (4)	0	17 (23)
Grade 3	0	0	0	0	0	1 (1)	1 (1)
Grade 4	0	0	0	0	0	0	0
							
*Late diarrhoea*							
Grade 2	2 (2)	2 (2)	2 (3)	1 (1)	2 (3)	0	7 (9)
Grade 3	0	0	0	0	0	0	3 (3)
Grade 4	0	0	0	0	0	2 (2)	0
							
*Total bilirubin*							
Grade 2	1 (1)	0	1 (2)	1 (3)	1 (1)	2 (2)	7 (13)
Grade 3	0	0	0	1 (1)	0	0	4 (4)
Grade 4	0	0	0	0	0	0	0
							
*Transaminases*							
Grade 2	0	0	0	0	0	0	3 (5)
Grade 3	0	0	0	0	1 (2)	0	3 (6)
Grade 4	0	0	0	0	0	0	0
							
*Hyponatremia*							
Grade 2	2 (3)	0	0	0	2 (2)	0	11 (17)
Grade 3	1 (1)	0	0	1 (1)	0	1 (1)	1 (1)
Grade 4	0	0	0	0	0	0	0

a Neutropenia grade.

**Table 6:** Objective response rates

		**CR**	**PR**	**CR+PR**		**PD**	
**Group**	**No. of evaluable patients**	**No.**	**No.**	**No.**	**%**	**No.**	**%**
Phase I							
60N	7	0	1	1	14.3	2	28.6
60P	5	0	1	1	20.0	2	40.0
65P	4	0	3	3	75.0	0	0
70P	3	0	0	0	0	1	33.3
75P	6	1	3	4	66.0	0	0
80P	2	0	1	1	50	0	0
Phase II	48	0	30	30	62.5 (47.3–76.0)^a^	7	14.6 (6.1–27.8)
Total	75	1	39	40	53.3 (41.4–64.9)	12	16.0 (8.5–26.3)

CR, complete response; PR, partial response; PD, progressive disease defined by WHO response criteria.

a 95% confidential interval.
